# Population attributable fractions for colorectal cancer and red and processed meats in Colombia - a macro-simulation study

**Published:** 2017-06-30

**Authors:** Esther de Vries, Doris C Quintero, Giana Henríquez-Mendoza, Oscar Fernando Herrán

**Affiliations:** 1 Department of Clinical Epidemiology and Biostatistics, Pontificia Universidad Javeriana. Bogotá. Colombia; 2 Grupo de Estudios Epidemiológicos y Salud Pública-FCV. Fundación Cardiovascular de Colombia. Bucaramanga. Colombia; 3 Subdirección de Investigaciones - Grupo Área de Salud Pública. Instituto Nacional de Cancerología. Bogotá. Colombia; 4 Escuela de Nutrición y Dietética. Universidad Industrial de Santander. Bucaramanga. Colombia

**Keywords:** Red meat, meat, processed meat, diet, risk, colorectal neoplasms, carcinogens, software, Colombia

## Abstract

**Aim:**

to estimate the population attributable risk of consumption of red and processed meat for colorectal cancer in Colombia.

**Methods:**

to model the expected incidence of colorectal cancer in the hypothetical situation of no red and processed meat consumption in Colombia, for the year 2010. A dynamic macrosimulation model, PREVENT 3.01, was used to integrate available cancer incidence, meat consumption prevalence and associated risk data and to evaluate the impact of eliminating red and processed meat from the Colombian diet on national colorectal cancer incidence.

**Results:**

Eliminating consumption of red meat altogether from the Colombian diet resulted in reductions in age-standardized colorectal cancer incidence, translating in reductions of 331 males (PAF 13%) and 297 female cases (PAF 10%). Eliminating processed meats had slightly stronger effects, with decreases of 362 males (PAF 14%) and 388 female cases (PAF 13%).

**Conclusions:**

A substantial proportion of the burden of colorectal cancer in Colombia can be attributed to the consumption of red and processed meat.

## Resumen

**Objetivo:**

estimar la fracción atribuible poblacional de consumo de carne roja y procesada para el cáncer colorrectal en Colombia.

**Métodos:**

modelación de la incidencia esperada de cáncer colorrectal en la situación hipotética de no consumo de carne roja y procesada en Colombia para el año 2010. Se utilizó un modelo dinámico de macrosimulación, PREVENT 3.01, para integrar los datos disponibles sobre incidencia de cáncer y evaluar el impacto de la eliminación de la dieta colombiana de la carne roja y procesada sobre la incidencia nacional de cáncer colorrectal.

**Resultados:**

La eliminación del consumo total de carne roja de la dieta colombiana dio lugar a reducciones en la incidencia estandarizada por edad de cáncer colorrectal, traduciéndose en la disminución de 331 casos en hombres (FAP 13%) y 297 en mujeres (FAP 10%). La eliminación de carnes procesadas tuvo efectos ligeramente más fuertes, con disminución de 362 casos en hombres (FAP 14%) y 388 en mujeres (FAP 13%).

**Conclusiones:**

Una proporción sustancial de la carga de cáncer colorrectal en Colombia puede atribuirse al consumo de carne roja y procesada.

## Introduction

Lifestyle factors, including diet, are important determinants of cancer risk ^1^. Recently, the International Agency for Research on Cancer (IARC) copied the conclusions previously made by the World Cancer Research Fund (WCRF). The conclusions are that the consumption of red meat is a probable carcinogen (class 2A) and that the consumption of processed meat is a carcinogen (class 1) ^2^. Red and processed meats particularly increase the risk of colorectal cancer. 

Several components of meats are thought to be responsible for the carcinogenic effects, including heme iron, and chemicals that form during meat processing or cooking such as N-nitroso compounds, heterocyclic aromatic amines and polycyclic aromatic hydrocarbons. Some of these chemicals are known or suspected carcinogens ^3^ ^,^ ^4^. However, it is not yet fully understood how cancer risk is increased by red meat or processed meat consumption ^4^.

According to the most recent estimates by the Global Burden of Disease Project, worldwide about 34,000 cancer deaths per year can be attributed to diets high in processed meat. The attributable risk proportion depends on local consumption patterns ^5^, and is unknown for Latin American countries. For policymakers and formulation of primary prevention measures, however, it is very useful to have local estimates of the proportion of (cancer) patients which can be attributed to exposure to certain risk factors. Therefore, in this project, we estimated the population attributable risk of colorectal cancer because of consumption of red meat and processed meats. We used to national detailed exposure information and estimates of cancer incidence, international risk association estimates and the macrosimulation program Prevent 3.01.

## Materials and Methods

In this study, we modelled the population attributable risk of colorectal cancer based on consumption of red and processed meats in Colombia for the year 2005. For the analysis, the following data were used: (i) sex-specific exposure patterns of red and processed meat; (ii) age- and sex-specific colorectal cancer incidence rates for the period 2007-2011; (iii) age- and sex-specific population size for the year 2010; (iv) risk functions for red and processed meat in relation to colorectal cancer.

We used PREVENT 3.01, a dynamic simulation model ^6^ , to integrate the data and to evaluate the impact of eliminating red and processed meat from the Colombian diet. 

### Datasources

i. Consumption of red and processed meats. ENSIN-2005 collected information on food consumption in children and adults (between 2 and 64 years), using 24-Hour Dietary Recall (R24H) methodology. To estimate the size of the portions consumed, standardized geometric food models were used. This guaranteed the precision of the portions consumed in the survey. The details of the methodology, collection, capture and cleaning of the databases been published previously ^7^. ENSIN-2005 was performed in 17,740 households, where 39,413 subjects responded to R24H, of which 49.4% were men ^7^.

Since the table of food composition used in ENSIN-2005 to translate consumption into macronutrients and micronutrients does not have the items "red meats" or "processed meats", it was necessary to prepare the original databases to estimate the consumption in grams (g) of these two items. For the above, first the different types of meats consumed were classified according IARC as "red meats", "processed meats" and "other" ^2^. The term "red meats" refers to beef, pork, lamb, veal and goat for domesticated animals. The term "processed meats" refers to meats preserved by smoking, fermentation, curing or salting, or addition de chemical preservatives. This classification was performed independently by two nutritionists; in cases when no consensus was established on the classification, the investigator (OFH) established the final classification. Subsequently, the consumption of red and processed meats in grams (g) was estimated for each subject, using FoodCalc, v.1.3 ^8^. Finally, the mean intake of red and processed meats (g) and their standard deviation was estimated by sex and age group (0-14; 15-44; 45-54; 55-64), constituting one of the inputs for the PREVENT software ^6^. Data processing was performed using Stata SE^®^ ^9^.

ii. Estimated age-and sex-specific colorectal cancer incidence rates for the period 2007-2011 were obtained from a publication by the National Cancer Institute of Colombia - which estimated subnational and national cancer incidence - by age group, crude and age-standardized, following a methodology derived from Globocan resulting in more detailed data than provided by Globocan 2012 ^10^. 

iii. The age- and sex specific projected population size for Colombia was obtained from Colombia's national statistics office Departamento Administrativo Nacional de Estadística (DANE).

iv. Risk functions for the relation between colorectal cancer and red and processed meat respectively were calculated based on the results of a large meta-analysis ^11^. We applied the following mathematical formula based on a consumption of 100 grams per day to calculate the reduction in risk for each reduction in steps of 20 grams per day (Table 1), assuming that Ln(RR(x))= intercept ^12^. Supplementary material summarizes the calculations. 

**Table 1:** Calculation of the risk functions

Relation between colorectal cancer risk and consumption of	Relative Risk		Intercept (lnRR)		Unit of change g/day		Change in risk with each unit change in Prevent	
	Males	Females	Males	Females	Males	Females	Males	Females
Red meat	1.17	1.17	-0.157	-0.157	20	20	0.002	0.002
Processed meat	1.35*	1.35*	-0.300	-0.300	20	20	0.002	0.002

*1.17 is the figure from the literature, corresponding to a consumption of 50 g per day of processed meat, transforming this risk to 100 g per day corresponds to 1.35.

### PREVENT model

To model the proportion of the colorectal cancer burden in Colombia attributable to consumption of red and processed meats, we used the simulation software PREVENT 3.01, adapted version for EUROCADET. A detailed description of the mathematical calculations of the PREVENT modelling software is given in a previous publication ^6^. In short, PREVENT 3.01 compares the projected future incidence of a disease without interventions (reference scenario) with the projected future incidence after reduction of exposure to risk factors (intervention scenarios) based on the standard formulae for estimation of population attributable fraction (PAF). Also PREVENT include a latency time in the model.

As we wanted an estimate of the PAF, we reduced the actual exposure to zero in the year 2010 for the intervention scenario, resulting in PREVENT calculating incidence in the reference ("real") scenario with the observed prevalence of red and processed meat consumption - versus incidence in the intervention scenario (with zero exposure) - resulting in the PAF. Since for PAF time lags in the effects of the risk factors are irrelevant in the PAF estimation, we did not model any latency or lag times. The only difference between the reference and intervention scenarios was therefore the reduction in the exposure.

Outcome measures were both absolute numbers and age-standardized rates of incident cases under the reference and intervention scenarios. Incidence rates were age-standardized using the SEGI world population and expressed per 100,000 person-years. The differences in absolute numbers were used to calculate the population attributable fraction under this model (i.e., proportion of colorectal cancers which would be avoided if red or processed meat were to be eliminated from the Colombian diet).

Sensitivity analysis: in order to estimate the range of uncertainty because of relative risks used, we used the lower and upper end of the confidence intervals of the meta-analysis in two sensitivity analyses, using the same prevalence and incidence data and the same modeling methods as in the main analyses (Table 2). 

**Table 2:** Results of sensitivity analysis assuming the confidence intervals for the attributable fraction.

Relation between colorectal cancer risk and consumption of	Relative Risk		Intercept (lnRR)		Unit of change g/day		Change in risk with each unit change in Prevent	
	Males	Females	Males	Females	Males	Females	Males	Females
Lower limit								
Red meat	1.05	1.05	-0.048	-0.048	20	20	0.002	0.002
Processed meat	1.27*	1.27*	-0.239	-0.239	20	20	0.002	0.002
Upper limit								
Red meat	1.31	1.31	-0.270	-0.270	20	20	0.002	0.002
Processed meat	1.45†	1.45†	-0.371	-0.371	20	20	0.002	0.002

*1.10 and † 1.28 are the figures from the literature, corresponding to a consumption of 50 g per day of processed meat in its lower and upper limits, transforming this risk to 100 g per day corresponds to 1.27 and 1.45.

#### Ethical Standards Disclosure

This article was based on secondary analysis of data on the incidence estimates for colorectal cancer produced by the Instituto Nacional de Cancerología de Colombia and Encuesta de Salud Nutricional de Colombia - ENSIN 2005. Ethical approval for this study was not required.

## Results

### Consumption of red and processed meats. 

In Colombia in 2005, we estimated the average daily consumption of red meats to be 62.4 g/day (95% CI: 61.4-63.3) for males and 55.0 g/day (95% CI: 54.2-55.7) for females. Mean daily consumption of processed meats was 58.3 g/day (95% CI: 56.4-60.1) for males and 50.7 g/day (95% CI: 49.1-52.3) for females. The consumption of red meat was highest in the age category 25-29 years (mean and SD of 70.4 g + 55.2), whereas the youngest age group of 2-4 years had the lowest consumption with 41.0 g + 26.7. Processed meat consumption was highest in ages 15-19 (mean consumption 61.6 g + 56.3) and lowest in the 55-59-year age group (42.9 g + 30.4). Supplementary material provides the estimated mean daily consumption for red and processed meat by sex and 5-year age group. 

**Figure 1:**
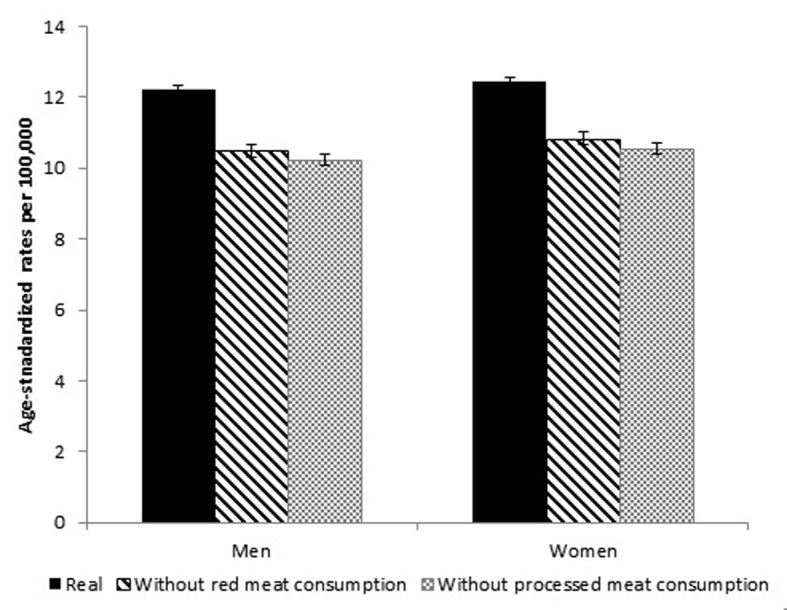
Age-standardized (SEGI standard population) colorectal cancer incidence rates estimated for Colombia, 2010, under the reference (real) and intervention scenarios

The intervention eliminating consumption of red meat altogether from the population resulted in reductions in age-standardized incidence rates from 12.2 to 10.5 and from 12.3 to 10.8 per 100,000 males and females, respectively, and to 10.3 and 10.6 for processed meats (Fig. 1).

In absolute numbers, the real incidence in 2010 was modelled to be 2,480 new male and 2,878 new female colorectal patients annually. In men, elimination of red meat consumption would avoid 331 patients and elimination of processed meats would avoid 362 new patients. For females, these numbers are 297 for red meat and 388 for processed meat elimination from the diet. This reduction translates into a population attributable fraction of 13% for males, and 10% for females for red meat consumption (average PAF 12%) and of 14% (males) and 13% (females) for processed meat consumption (average PAF 14%). Table 3 shows the effect of the risk function estimates, based on the upper and lower limits of the risk estimates, the proportion of new cases avoided under the scenario of elimination of red meat consumption, between 7 and 20% of male, and between 5 and 15% of female cases would be avoided. For elimination of processed meat consumption, this interval is 10-19% for males and 9-18% for females. 

**Table 3:** Population attributable fraction in absolute numbers and proportion of avoidable colorectal cancers by eliminating red and processed meat consumption

Risk factor	Sex	Colorectal cancer incidence 2010 (reference)	Colorectal cancer incidence 2010 under simulation* (intervention)	95% CI of optimal incidence in absolute numbers†		Proportion avoidable colorectal cancers*	95% CI of proportion avoidable colorectal cancers†	
		Numbers	Numbers	Lower limit	Upper limit	%	Lower limit %	Upper limit %
Red meat	Men	2,480	2,149	1,992	2,312	13	7	20
	Women	2,878	2,581	2,439	2,727	10	5	15
Processed meat	Men	2,480	2,118	2,003	2,236	15	10	19
	Women	2,878	2,490	2,363	2,618	14	9	18

*under elimination of red or processed meat consumption

†Based on the lower and upper end of the 95% confidence intervals of the meta-analyses of the relative risks for colorectal cancer for red and processed meat consumption

## Discussion

This modeling exercise, based on existing data sources, shows that an expected 11.9% of colorectal cancers occurring in the Colombian population is attributable to the consumption of red meats, whereas 13.9% is attributable to processed meats. Not many studies have estimated population attributable risks or fraction for the consumption of red and processed meat, one Australian study estimated that 18% of colorectal cancer occurring in Australians in 2010 were attributable to red/processed meat consumption, but this study did not differentiate between red and processed meat consumption ^13^. Similar figures for the UK were estimated to be 21.1% ^14^. Of course, this fraction is highly dependent on both the incidence of the disease, but more importantly, on the meat consumption patterns of the population. 

Colorectal cancer in Colombia seems to be on the rise, based on trends with 2% annual increase in mortality figures ^15^ and observations since 1962 from the population-based cancer registry of the city of Cali ^16^ ^,^ ^17^. Important risk factors for cancer include, besides the consumption of meat, harmful use of alcohol, diabetes, low fruit and vegetable consumption, low physical activity, y tobacco consumption ^18^. A low participation rate of colorectal cancer screening also influences the risk substantially ^19^. The observed increases in colorectal cancer incidence are unlikely to be due to changes in alcohol consumption, which according to WHO has been flat over the past decades in Colombia ^20^. Trends in levels of physical activity are unclear; whereas leisure time physical activity decreased, active ways of transportation (walking, biking), increased in the Colombian population between 2005 and 2010 ^21^. In general, levels of physical activity are relatively low in Colombia. Despite the enormous offer and diversity of fruits and vegetables in this tropical country, the consumption of fruits is very low with a median of 88 g/day (mode 12 g/day), and the vegetable consumption is even lower: 45.75 g/day (mode 14.5 g/day) ^22^. Trends in diabetes are increasing, according to unofficially published figures by the Colombian Observatory for Diabetes ^23^.

The consumption prevalence on which we based the figures for the population and incidence data of 2010 was for 2005. We modelled this delay because exposure to dietary carcinogens is unlikely to have a very short term effect and therefore we believe the five-year gap between prevalence and incidence data does not cause any problem for these estimates.

### Strengths and weaknesses

As in any modeling exercise, the results depend entirely on the input data and assumptions used, most importantly the cancer incidence, consumption prevalence and risk function information. Even though all three sources have their levels of uncertainty, they were based on the best available information. Our incidence data were estimates based on modeling using data from 4 high quality regional population based cancer registries as well as official national mortality data, which methods are described in detail elsewhere ^10^. Although no national cancer registry is available, we believe these figures are close to represent the actual colorectal cancer incidence in Colombia. We used national representative studies to estimate the red and processed meat consumption in Colombia and were able to differentiate between the two types of meat consumption. 

Sensitivity analysis using the extremes of the confidence intervals for the risk functions resulted in a range of likely estimates, which were wider for males and for red meat because of the greater spread of consumption pattern combined with wider confidence intervals of the relative risks. We did not perform any sensitivity analysis for the incidence and prevalence data, since their confidence intervals were rather narrow, therefore they will result in minimum variation in the final estimates.

## Conclusion

Colorectal cancer incidence could be reduced if interventions were made to reduce red and processed meat consumption, because around 12% of colorectal cancer cases in Colombia can be attributed to red meat consumption, and 14% to processed meat consumption. The growing burden of this cancer can be curbed by reducing this consumption as well as focusing on increasing dietary fiber consumption (eg. fruit and vegetable) as well as physical activity and increase participation in screening programs. 

## Supplementary material

**Table 4:** Age groups by sex and average amounts of consumption of red meats and processed meats.

Age group	Red meat						Processed meats					
	Men			Women			Men			Women		
	N (gr)	Mean (gr)	Sd (gr)	N (gr)	Mean (gr)	Sd (gr)	N(gr)	Mean (gr)	Sd (gr)	N (gr)	Mean (gr)	Sd (gr)
2-4	1,181	41.63	25.74	1,151	40.34	27.69	279	44.52	37.11	285	42.02	34.97
5-9	2,090	49.57	33.72	2,137	47.87	31.66	755	51.31	46.97	667	49.10	46.13
10-14	2,358	57.98	43.35	2,271	57.38	39.96	802	61.97	59.22	779	56.21	49.42
15-19	2,018	69.13	53.45	1,966	60.75	42.25	619	70.17	64.19	644	53.43	46.07
20-24	800	77.03	58.27	853	62.62	44.19	261	54.52	46.25	248	50.61	42.00
25-29	520	76.77	61.81	580	64.74	47.82	137	66.24	58.87	138	48.99	41.85
30-34	444	79.79	67.22	476	60.16	44.09	108	63.45	68.75	103	46.09	46.47
35-39	423	78.02	55.70	461	56.56	37.71	102	54.98	60.58	110	47.24	40.41
40-44	384	77.86	58.97	415	58.35	42.25	79	49.07	40.34	87	46.07	38.40
45-49	365	71.53	53.74	339	52.86	34.62	56	55.82	47.90	64	48.56	49.83
50-54	259	71.12	60.20	278	54.20	36.42	40	53.15	44.50	49	40.33	39.53
55-59	176	75.22	58.72	201	53.48	34.26	22	47.27	34.20	21	38.29	25.83
60-64	162	65.18	48.70	146	51.03	36.58	27	46.72	37.28	27	46.31	48.29
